# PCL/Col I-based magnetic nanocomposite scaffold provides an osteoinductive environment for ADSCs in osteogenic cues-free media conditions

**DOI:** 10.1186/s13287-022-02816-0

**Published:** 2022-04-04

**Authors:** Hadi Sadeghzadeh, Ahmad Mehdipour, Hassan Dianat-Moghadam, Roya Salehi, Ali Baradar Khoshfetrat, Ayla Hassani, Daryush Mohammadnejad

**Affiliations:** 1 Department of Tissue Engineering, Faculty of Advanced Medical Sciences, Tabriz University of Medical Sciences, Tabriz, Iran; 2 Department of Genetics and Molecular Biology, School of Medicine, Isfahan University of Medical Sciences, Isfahan, Iran; 3 Department of Medical Nanotechnology, Faculty of Advanced Medical Science, Tabriz University of Medical Science, Tabriz, Iran; 4 Chemical Engineering Faculty, Sahand University of Technology, 51335-1996 Tabriz, Iran; 5 Department of Anatomical Sciences, Faculty of Medicine, Tabriz University of Medical Sciences, Tabriz, Iran

**Keywords:** Scaffold, Type I collagen, Magnetic nanoparticles, Adipose-derived stem cells, Differentiation, Osteogenic cues-free media

## Abstract

**Background:**

The bone tissue engineering (BTE) approach has been introduced as an alternative to conventional treatments for large non-healing bone defects. Magnetism promotes stem cells' adherence to biocompatible scaffolds toward osteoblast differentiation. Furthermore, osteogenic differentiation media are expensive and any changes in its composition affect stem cells differentiation. Moreover, media growth factors possess a short half-life resulting in the rapid loss of their functions in vivo. With the above in mind, we fabricated a multilayered nanocomposite scaffold containing the wild type of Type I collagen (Col I) with endogenous magnetic property to promote osteogenesis in rat ADSCs with the minimum requirement of osteogenic differentiation medium.

**Methods:**

Fe_3_O_4_ NPs were synthesized by co-precipitation method and characterized using SEM, VSM, and FTIR. Then, a PCL/Col I nanocomposite scaffold entrapping Fe_3_O_4_ NPs was fabricated by electrospinning and characterized using SEM, TEM, AFM, VSM, Contact Angle, tensile stretching, and FTIR. ADSCs were isolated from rat adipose tissue and identified by flow cytometry. ADSCs were loaded onto PCL/Col I and PCL/Col I/Fe_3_O_4_-scaffolds for 1–3 weeks with/without osteogenic media conditions. The cell viability, cell adhesion, and osteogenic differentiation were evaluated using MTT assay, SEM, DAPI staining, ALP/ARS staining, RT-PCR, and western blotting, respectively.

**Results:**

SEM, VSM, and FTIR results indicated that Fe_3_O_4_ was synthesized in nano-sized (15–30 nm) particles with spherical-shaped morphology and superparamagnetic properties with approved chemical structure as FTIR revealed. According to SEM images, the fabricated magnetic scaffolds consisted of nanofiber (500–700 nm). TEM images have shown the Fe_3_O_4_ NPs entrapped in the scaffold's fiber without bead formation. FTIR spectra analysis confirmed the maintenance of the natural structure of Col I, PCL, and Fe_3_O_4_ upon electrospinning. AFM data have shown that MNPs incorporation introduced stripe-like topography to nanofibers, while the depth of the grooves has decreased from 800 to 500 nm. Flow cytometry confirmed the phenotype of ADSCs according to their surface markers (i.e., CD29 and CD105). Additionally, Fe_3_O_4_ NP improved nanocomposite scaffold strength, wettability, porosity, biocompatibility and also facilitates the ALP activity, calcium-mineralization. Finally, magnetic nanocomposite scaffolds upregulated osteogenic-related genes or proteins’ expression (e.g., Col I, Runx2, OCN, ON, BMP2) in seeded ADSCs with/without osteo-differentiation media conditions.

**Conclusions:**

Together, these results indicate that Fe_3_O_4_ NPs within the natural structure of Col I increase osteogenic differentiation in osteogenic cues-free media conditions. This effect could be translated in vivo toward bone defects healing. These findings support the use of natural ECM materials alongside magnetic particles as composite scaffolds to achieve their full therapeutic potential in BTE treatments.

**Graphical Abstract:**

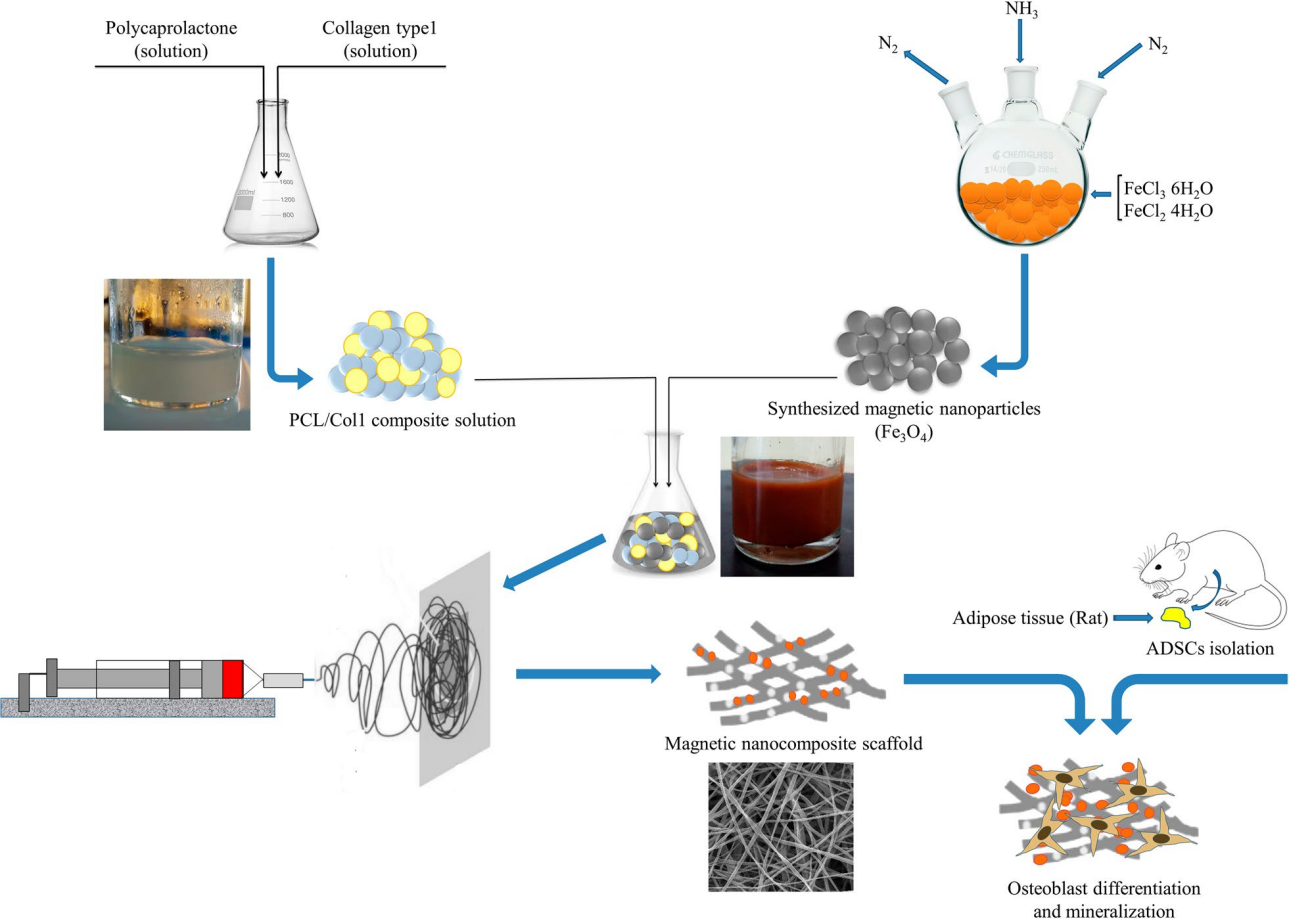

## Introduction

Bone defects caused by trauma or disease remain a clinical challenge for public health worldwide, and the deficiencies within the bone grafts persuaded researchers to explore bone tissue regeneration, repair, and engineering (BTE) [1, 2]. Commonly, BTE has been developed to mimic autologous bone grafts involving living cells, scaffolds, and growth factors [3]. Adipose-derived mesenchymal stem cells (ADSCs) are multipotent cells that can differentiate into specific lineages including chondrocyte, osteoblast, myoblast, and neural cells. ADSCs have shown a high attachment rate to scaffold material, and a high differentiation capacity into the osteogenic lineage. ADSCs secret growth factors contribute to the bone tissue remodeling and angiogenesis [4]. Compared to bone marrow-derived MSCs (BMSCs), ADSCs can be obtained in a high ratio, are easily available and harvestable, and have low sensitivity to senescence [4].

Commonly, osteogenic media provide cytokines and growth factors for BTE. However, these media are expensive and it’s any compositional changes affect ADSCs differentiation into untargeted cells. Moreover, media growth factors possess a short half-life resulting in the rapid loss of their functions in vivo [5]. Thus, developing a cost-effective and modified scaffold with the minimum requirement of osteo media is demanded.

The scaffold plays a core role in artificial bone. It should mimic physio-chemical properties of the native tissue extracellular matrix (ECM), support cell adherence, growth, and osteogenic differentiation [6]. Polycaprolactone (PCL) is an FDA-approved polymer with suitable biocompatibility, thermal stability, and hydrophobic property-providing stem cell adhesion, making it fit for BTE [7]. Slow degradation of PCL is challenging in soft tissue engineering but makes it an ideal material for hard tissues such as bone [7]. Collagen type I (Col I) is the main organic component of natural bone tissue ECM. Hence, Col I is one of the preferred ECM proteins for application in BTE scaffolding. Natural Col I presents RGD peptide that enhances the biocompatibility and produces conditions for seeding, proliferation, and differentiation of seeded cells in BTE [7, 8]. The electrospinned PCL scaffold with high porosity facilitates cell proliferation and reorganization, and nutrient exchange. However, high porosity reduces the PCL mechanical strength which can be improved by controlling the ratio of pores and method of fabrication and preparing in multilayered three-dimensional (3D) structure [9].

Current studies have discovered that the cellular adhesion on the polymeric scaffold can be reinforced by surface coating with magnetic nanoparticles (MNPs) to produce nano-scaled magnetic stimuli [10, 11]. MNPs increase surface roughness and area, thus augmenting adhesion loci for stem cells, and also introduce osteoconductive and osteoinductive effects to accelerate bone defect healing [12]. However, they decrease ADSC's ability to differentiate into adipogenic lineage [13]. The superparamagnetic iron oxide nanoparticles (SPIONs) enhance the alkaline phosphatase (ALP) activity and calcium content in a rat model that indicate its potentials to induce osteogenesis and angiogenesis [14]. SPIONs also induce MAPK signaling pathway to promote osteogenic differentiation of BMSCs [15]. SPIONs also upregulate the *INZEB2*, an important long noncoding RNA in MSCs, which is necessary for the sustained osteogenesis process [16]. Compared to PCL scaffold, MNPs incorporated PCL scaffolds are favorable tissue biocompatible scaffold that enhances cell adhesion, proliferation, and mineralization and induce neoangiogenesis supporting bone repair and regeneration in vivo [11]. Moreover, MNPs introduce the ability of self-reinforcement in PCL to achieve a better strength. Loading the MNPs into the scaffold can be performed by electrospinning and layer-by-layer (LbL) methods. Electrospinning provides a high surface area/volume ratio, controllable fiber diameters, high porosity and permeability [12]. LbL assembly formed multilayer films of scaffold that improve stem cell attachment and proliferation. Moreover, the combination of electrospinning and LbL assembly enhances the integrity of MNP-scaffolds and also promotes the osteogenic differentiation of ADSCs [17]. Collectively, the magnified scaffolds can be considered as a promising ECM in BTE applications.

Here, we investigated the application of electrospinning to fabricate the multilayered magnetic scaffolds containing PCL, Col I, and Fe_3_O_4_-MNPs (or SPIONs). We hypothesize that the fabricated scaffold may enhance the ADSCs attachment and synergize the osteogenic effect. We examined our hypothesis by carrying out related cellular, molecular, and characterizing experimental tests in vitro. This platform may improve designing the next-generation scaffolds with intrinsic magnetic stimuli for BTE in future works.

## Materials and methods

### Synthesis and characterization of magnetite nanoparticles

Superparamagnetic iron oxide nanoparticles (Fe_3_O_4_) were synthesized using an improved chemical co-precipitation method. According to this method, 3.1736 g of Ferrous chloride tetrahydrate (FeCl_2_·4H_2_O) and 7.5684 g of ferric chloride hexahydrate (FeCl_3_·6H_2_O) were dissolved in 320 mL of deionized water. (Fe^2+^/Fe^3+^  = 1/1.75). The mixed solution was stirred under nitrogen at 80 °C for 1 h. Then, NH_3_·H_2_O 40 mL was injected into the mixture rapidly, stirred under nitrogen for another hour, and then cooled down to room temperature. The precipitated particles were washed five times with hot water and separated by magnetic decantation. Finally, the magnetic nanoparticles were dried under vacuum at 70 °C.

Morphology and size of the MNPs were determined by field emission scanning electron microscopy (FE-SEM) using a model S-3000H microscope (Hitachi). The magnetization curves of the samples were measured with a vibrating sample magnetometer (VSM; Lakeshore Cryotronics, Westerville, OH, USA) at room temperature. The chemical composition of the samples was detected by Fourier transform infrared spectroscopy (FTIR, Perkin-Elmer) between 4000 and 400 cm^−1^.

### Preparation of magnetic nanocomposite scaffolds

**Fig. 1:**
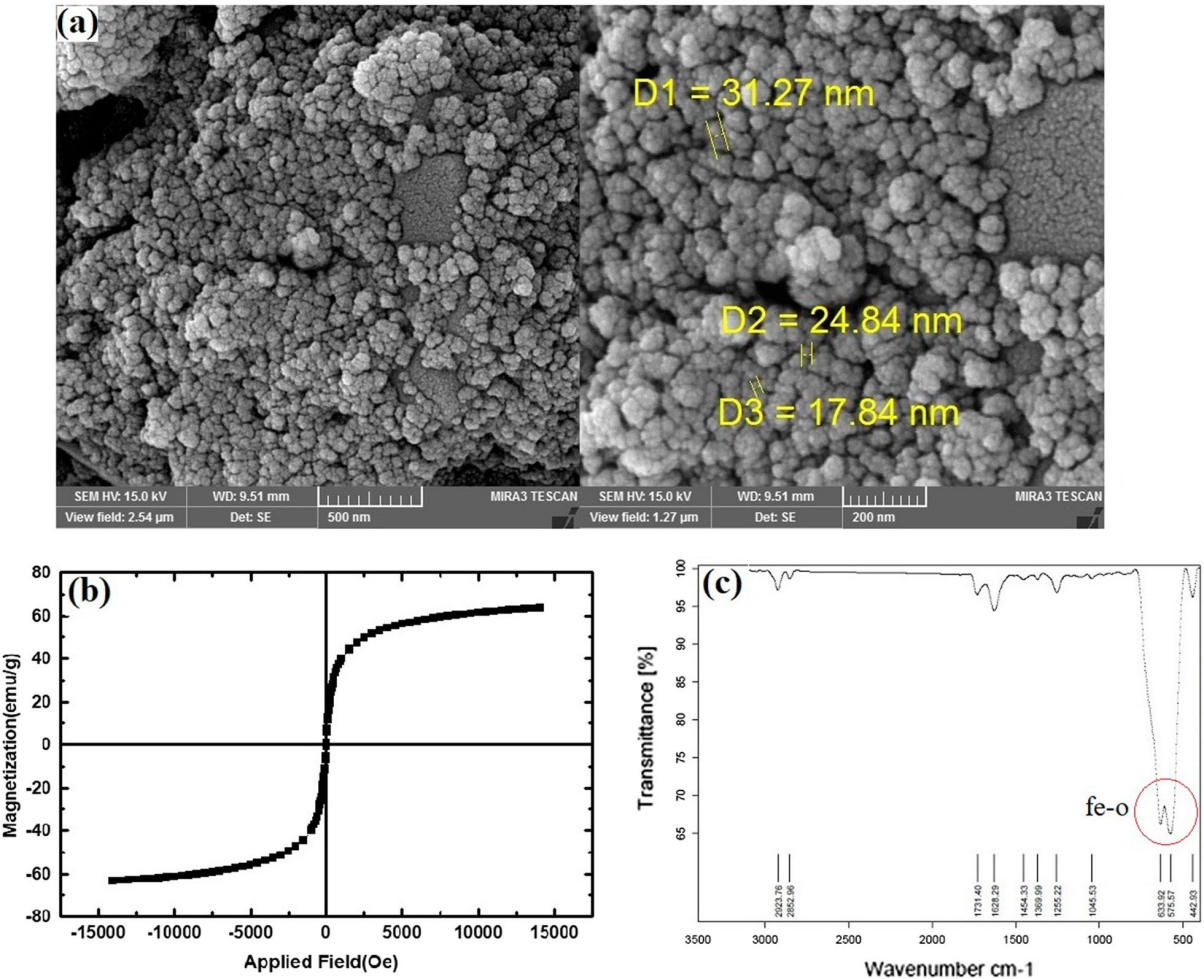
Morphology and characterization of MNPs synthesized via co-precipitation method of Fe_3_O_4_. **a** SEM images of MNPs at different resolutions (sized 15–30 nm). Scale bars equal 500 and 200 nm, respectively. **b** Magnetic properties of the MNPs analyzed by a VSM. (**c**) FTIR spectra of the MNPs in the range of 570–590 cm^−1^

A schematic image of the preparation of nanocomposite scaffold is shown in Fig. 1. At first, the PCL pallet (~ 80 kDa, Sigma-Aldrich, USA) was dissolved in glacial acetic acid at 10%w/v. Next, the type I collagen (Col I) (SBPE company, Tabriz, Iran) was dissolved in the NaAc/HAc solution (0.7 g CH_3_COONa-3H_2_O in 3 mL CH_3_COOH) at 10%w/v. Then, PCL/Col I solution (9:1 ratio) was prepared. MNPs suspension dispersed by water-bath ultrasonication (Jikang Ultrasonic Equipment Co., Ltd, China) for 10 min and then was added to the PCL/Col I solution at 5 wt%.

The nanocomposite scaffolds (PCL/Col I with and without fe_3_o_4_ magnetic nanoparticles) were prepared using electrospinning. In brief, the solutions were filled to syringe pump with a metal needle (internal diameter = 0.4 mm; Jianpai, Jintan, China) and the needle was connected to a high-voltage power supply (21 kV), and the distance between tip-to-collector was kept at 15 cm and injection rate was 1 ml/h to form the nanofibrous structure on the aluminized collecting plate with the drum rotating speed (150 rpm). The multilayered fabricated scaffold was dried for 72 h before use. All experiments were carried out at room temperature.

### Characterization of nanocomposite scaffolds

The fiber’s size, pore structure, and surface morphology of the nanocomposite scaffolds were observed by FE-SEM. Transmission electron microscopy (TEM; Tecnai G2 Spirit Bio TWIN, FEI, Hillsboro, OR) was used to examine the nanoscale morphology of nanocomposite scaffold. The chemical bond of the scaffolds was analyzed by Fourier transformed infrared spectroscopy (FTIR; PerkinElmer, USA). Surface water contact angles of the scaffolds were measured using a contact angle meter (JC2000C2, Shanghai Zhongchen Powereach Company, China). The magnetic properties of the nanocomposite scaffold were evaluated by a VSM at room temperature. The surface topography and roughness of samples were analyzed by atomic force microscopy imaging (AFM Agilent 5500, Chandler, AZ), and then, Nanosurf Mobile-S software was used to process the obtained images. Tensile strength tests were carried out to determine the mechanical strength of the electrospun nanocomposite scaffolds (Instron 3344 universal testing instrument). After the tensile load was applied at a cross-head speed of 5 mm/min with a 30 mm gauge length, the stress–strain curves were recorded from the test. The specimens were 0.05 mm in thickness and 25 mm in diameter.

### ADSCs isolation and characterization

In this study, mesenchymal stem cells were isolated from the abdomen adipose tissue of rats (Wistar albino, male, 250–300 g, 6–8 weeks old). After the xylazine (Richter Pharma AG, Austria) (10 mg/kg) and ketamine (Richter Pharma AG) (50 mg/kg) anesthesia of rats, adipose tissue was isolated from the rat abdomen and washed with Phosphate Buffered Saline (PBS, Hyclon, Logan City, UT, USA) containing 5% Penicillin Streptomycin (Pen-Strep, Hyclon, America). Then, the isolated adipose tissue was minced and digested with 1 mg/mL of collagenase type I (Sigma, St., Louis, MO, USA) at 37 °C for 70 min. After filtration through a 100-mesh cell strain, the filtrate was centrifuged for 5 min to collect adipose-derived mesenchymal stem cells (ADSCs). The pellet was re-suspended into Dulbecco’s Modified Eagle Media (DMEM, Gibco) complete medium (DMEM basic medium with 10% fetal bovine serum, 1% Pen-Strep, and 1% L-glutamine) and was transferred to T25 cell culture flask then placed in an incubator under a humidified atmosphere of 5% CO2 in air at 37 °C. The flow cytometry analysis was used to detect the cultured cells’ surface markers (CD29, CD105, CD45, and CD34). Multilineage differentiation potential (Adipogenic, chondrogenic, and osteogenic) of isolated ADSCs was assessed using Oil Red O, Alcian blue, and Alizarin Red S staining in vitro at P3. To assess adipogenic and osteogenic differentiation, ADSCs (1 × 10^4^ cells/cm^2^) were cultured in the DMEM supplemented with 10% FBS and 1% penicillin/streptomycin and were incubated at 37 °C in a 5% CO2 humidified atmosphere for 24 h. After incubation, the basal media were replaced with the adipogenic or osteogenic differentiation medium. ADSCs were cultured in an osteoinductive medium containing high glucose DMEM, FBS 10%, dexamethasone (10^–7^ M), beta-glycerol-phosphate (10 mM), and ascorbic acid bi-phosphate (50 μg/mL) for 21 days. The differentiation medium was changed twice a week as previously described [18]. Then, Alizarin Red S was used to detect calcium deposition in differentiated ADSCs. To induce adipogenic differentiation, cells were cultured for 16 days in an adipo-inductive medium (containing high glucose DMEM, 10% FBS, 0.5 mM IBMX, 250 nM dexamethasone, 66 nM insulin, and 0.2 mM indomethacin). Oil Red O staining was used to detect the adipogenic differentiation of ADSCs. For chondrogenic differentiation, 2 × 10^5^ cells were re-suspended and centrifuged at 600 g for 5 min in a 15 ml conical tube. The procedure was followed by the addition of 1 ml of chondro-inductive medium (high glucose DMEM, 10% FBS, 10 ng/mL TGF-β, 50 μg/mL ascorbic acid bi-phosphate, 10^–7^ M dexamethasone, and 10 ng/mL bFGF) in the conical tube containing cell pellet. Cells were kept at standard condition inside the chondro-inductive medium. The exhaust medium was replenished twice per week. After the completion of the incubation period, the supernatant was discarded and 200 µl paraformaldehyde (4% w/v) was overlaid on cell pellet and maintained at 4 °C overnight. Paraffin-embedded samples were cut into 5-µm-thick sections and stained with Alcian blue solution to determine glycosaminoglycans and mucopolysaccharides in ADSCs exposed to differentiation medium. Slides were visualized and imaged using Olympus microscopy.

### In vitro cell assays

#### Nanocomposite scaffold biocompatibility

MTT assay analysis was used to examine the viability and compatibility of ADSCs loaded into scaffolds (PCL/Col with and without MNP). ADSCs were used in passages 3–5. For MTT assay, cells were grown in DMEM supplemented with 10% FBS and 1% penicillin/streptomycin and were incubated at 37 °C in a 5% CO2 humidified atmosphere. The ultraviolet irradiation (12 h) was used to sterilized the scaffolds. Next, samples were placed in 96-well culture plates (SPL, Korea), followed by seeding 0.5 × 10^4^ ADSCs onto each well. The cells grown in media without a scaffold served as control. After 2,5, and 7 days of incubation, the media of each well were removed carefully and replaced with MTT solution (2 mg/mL MTT dissolved in PBS), which was diluted in a media. Then, the plates were covered with aluminum foil and incubated for 4 h. After removing the MTT solution, 200 μl of pure DMSO and 25 μl Sorensen’s glycine buffer were added to each well. Finally, the UV absorbance of each well was measured at 570 nm by ELISA-reader. All the experiments were performed in triplicates.

#### Morphology and adhesion of ADSCs onto nanocomposite scaffolds

SEM observation was used to assess the morphology and adhesion of ADSCs seeded onto scaffolds. After 48 h of incubation, the samples were washed with PBS and fixed using 4% glutaraldehyde for 30 min. In the following, the samples were dehydrated and dried for 24 h. After the sample’s surface was coated with gold, cells morphology was observed using FE-SEM imaging.

4′,6-diamidino-2-phenylindole (DAPI) staining was used to detect ADSCs seeded onto scaffolds. Briefly, 24 and 72 h after 0.7 × 10^4^ cells seeding, samples were collected and washed thrice with PBS. Next, the cell scaffolds were fixed with 2% paraformaldehyde solution (diluted in PBS) for 10 min at room temperature. Subsequently, the samples were washed 2–3 times with PBS and the permeabilization buffer (10% triton x-100) was added to each sample for 15 min at room temperature. After samples washing thrice with PBS, 300 µL of DAPI stain solution (300 nM) was added to each sample, and next, the stained samples were covered with aluminum foil. After 5 min incubating at room temperature, the stain solution was removed and washed 2–3 times with PBS. Subsequently, stained samples were visualized using by Olympus BX53 fluorescence microscope at 405 nm with a DP80 CCD camera (Olympus).

### Osteoblast differentiation assays

ADSCs were used in passages 3–5 for differentiation assays tests. DMEM culture media were supplemented with 50 μM ascorbate-2-phosphate, 10^−7^ M dexamethasone, and 10 mM β-glycerol phosphate (Sigma-Aldrich) to form osteogenic medium. The cell scaffolds were cultured with and without osteogenic medium in 48- and 6-well cell culture plates for subsequent experiments. The cell scaffolds were maintained by changing fresh medium every 2–3 days.

Alkaline phosphatase (ALP) and Alizarin red S (ARS) staining were carried out to qualitative investigation of osteogenic differentiation of the ADSCs seeded on scaffolds. After 7,14, and 21 days of incubation, the medium was removed and the samples were washed twice with PBS. Next, the samples were fixed with 4% paraformaldehyde for 15 min. For the ALP staining, the samples were washed twice with distilled water and then permeabilized with 0.2% Triton X-100 for 10 min. After the samples rinsing with distilled water, the Naphthol-AS-BL alkaline solution mixture (Sigma-Aldrich) was added to each sample and incubated at 37 °C for 30 min. The color change of the reaction products was visualized with a light microscope. Separately, for calcium-rich deposits staining on the scaffolds (ARS staining), the fixed samples were washed and stained with 40 mM ARS solution (pH 4.1–4.3) for 10 min. Next, the scaffolds were rinsed with distilled water and visualized with a light microscope (Olympus IX71).

After 21 days of cells seeding, total RNA was extracted with TRIzol reagent (Invitrogen, Carlsbad, CA). Reverse transcription polymerase chain reaction (RT-PCR) analysis was used to evaluate osteogenic differentiation-related genes including type 1 Collagen (Col 1), runt-related transcription factor 2 (Runx2), and osteocalcin (OCN). The glyceraldehyde 3-phosphate dehydrogenase (GAPDH) was used as a housekeeping gene. The primer's sequences are listed in Table1. Total RNA purity and concentration were determined by a NanoVue Plus spectrophotometer (GE Healthcare, Piscataway, NJ). 2 μg of isolated RNAs from each sample was used to synthesized complementary DNA (cDNA) by PrimeScript RT reagent kit (Takara Bio Co., Ltd., Otsu, Japan). Quantitative RT-PCR (qRT-PCR) reactions were carried out by RT-PCR System (Roche LightCycler® 96 Instrument): A 5 min denaturation step at 95 °C was followed by 40 cycles of 20 s at 95 °C and 60 s at 61 °C. The relative gene expressions were evaluated using a 2^−ΔΔCt^ method, which was normalized by the cycle threshold (Ct) of the housekeeping gene GAPDH in triplicate.

**Table 1:** Real-time polymerase chain reaction primers used in this study

Gene	Forward sequence	Reverse sequence
*COL 1*	5′- TGAGACAGGCGAACAAGGTGAC -3′	5′- GGACCAGCAGGACCACTATCG -3′
*RUNX2*	5′- TTCGTCAGCGTCCTATCAGTTCC -3′	5′- CCATCAGCGTCAACACCATCATTC -3′
*OCN*	5′′-ACCCTCTCTCTGCTCACTCTGC-3′	5′-CCTTACTGCCCTCCTGCTTGG-3′
*GAPDH*	5′- CCTGCACCACCAACTGCTTA -3′	5′- AGTGATGGCATGGACTGTGG -3′

### Western blotting

Western blotting was performed to investigate the master proteins involved in the regulation of osteogenesis. Cell scaffolds were lysed with buffer containing 50 mM Tris–HCl (pH 8), 5 mM EDTA, 1% Triton X-100, 150 mM NaCl, 10 mM NaF, 1 mM sodium orthovanadate and 1 mM protease cocktail inhibitor (Roche) and incubated at 4 °C for 1 h. The cell lysates were ultrasonicated and centrifuged at 12,000 × g for 20 min at 4 °C. Then, the total protein concentration was determined using Nanodrop®. The protein extracts of each sample were electrophoresed by 10% SDS-PAGE and transferred onto nitrocellulose membranes. After blocking non-specific binding sites with TBS and 5% non-fat dry milk for 2 h, the membranes were incubated at 4 °C overnight with primary antibodies (Santa Cruz Biotechnology, Santa Cruz, USA) against Col I, Runx2, OCN, ON and BMP2. Subsequently, the samples were incubated with a horseradish peroxidase-conjugated secondary antibody (Santa Cruz Biotechnology, Santa Cruz, USA) for 1 h at room temperature. The osteoblast cell lines (UMR-106 cell lines, National Cell Bank of Iran, pasture institute of Iran, Tehran) were used as a positive control. Finally, immunoreactive bands were detected by using ECL reagent (BioRad). The density of each band was determined using ImageJ software (version 1.4).

### Statistical analysis

In this study, graph Pad Prism software version 9 (GraphPad Software, Inc., San Diego, CA) was used to analyze the experimental data. The results were expressed as mean ± standard deviation. Statistical analyses were performed by one-way analysis of variance (ANOVA) followed by multiple comparison Tukey’s test. Results were considered significant (*p* < 0.05) and highly significant (*p* < 0.01), (*p* < 0.001), (*p* < 0.0001).

## Results and discussion

### Physicochemical properties of magnetic NPs/scaffold

Synthesized Fe_3_O_4_-MNPs were observed by SEM, and the spherical-shaped MNPs were produced well with uniform sized (~ 20 nm) and homogeneously distributed (Fig. 1a). These MNPs possess acceptable size (i.e., below 30 nm) and are approved by FDA for biomedical applications such as BTE [19]. Moreover, VSM results confirmed the superparamagnetic properties of synthesized Fe_3_O_4_-based MNPs in response to the magnetic field [5] (Fig. 1b). As expected, FTIR analysis showed a significant peak around 580 cm^−1^ (Fig. 1c), which is attributed to the stretching vibrations from Fe–O and confirms the existence of NPs with magnetite core [20].

**Fig. 2:**
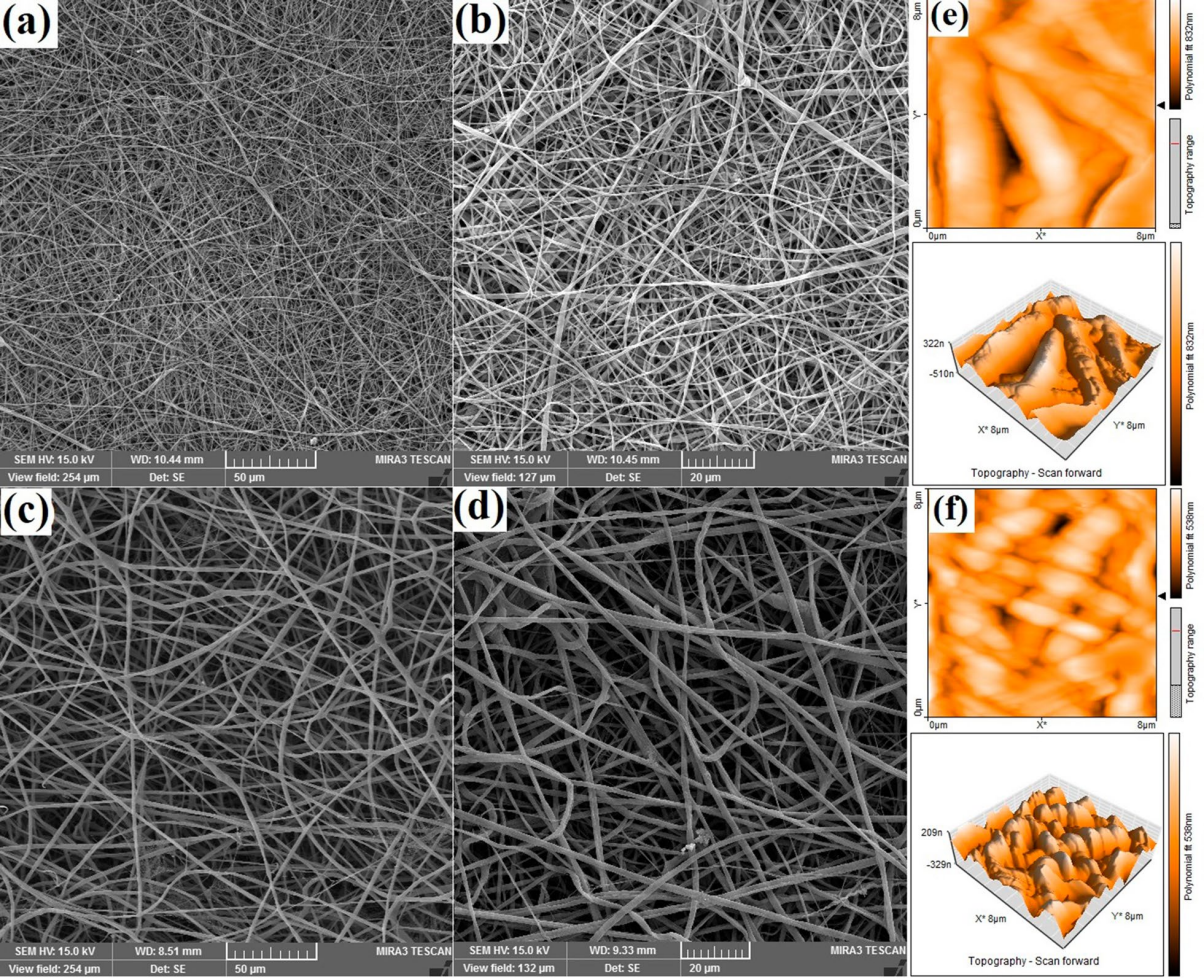
Morphology and surface characterization of fabricated scaffolds. **a–c** SEM view of MNPs-free PCL/Collagen scaffolds at different resolutions (sized 200–500 nm). **d–f** SEM view of MNPs + PCL/Col I scaffolds at different resolutions. Scale bars equal 50, 20 and 1 µm, respectively. 2D and 3D topography of nanocomposite scaffolds whiteout (**g**), and with MNPs (**h**) via AFM at room temperature

The morphological analyses of PCL/Col I scaffold with and without MNPs are assessed through SEM, TEM, and AFM. In the case of the PCL/Col I scaffold (Fig. 2a–c), the SEM result has shown that the composite was micrographs of the sintered 2D scaffold which has interconnected porosity. The random-sized pores including macropores (70–150 μm) and micropores (1–7 μm) observed in SEM images are suitable for cell infiltration [21] (Fig. 2a). This parameter mimics the natural bone microstructure supporting cell attachment and spreading, nutrient delivery, and the formation of internal mineralized [22, 23]. The SEM also has shown that the fibers are homogeneously distributed, and no bead observed under the incorporation of MNPs in optimal concentration (Fig. 2a–f). Additionally, SEM results have revealed the nanocomposite structure containing the nanofiber’s size between 300–500 nm and 50–150 nm. Incorporated MNPs increase the diameter of the fibers by 200 to 300 nm with no effects on the fiber’s structure (Fig. 2d–f).

AFM was used to further characterize the 3D surface morphology of the nanocomposite scaffolds. The topography of fibers introduced stripe-like and wavy patterns to the surface of the MNPs-free scaffolds (Fig. 2g), which those waves were increased over MNPs incorporation, while the depth of the grooves has been decreased from 800 to 500 nm (Fig. 2h). This modification all augmented surface roughness and extended surface area to nanocomposite magnetic scaffold providing cellular adherence as well as differentiation [24].

**Fig. 3:**
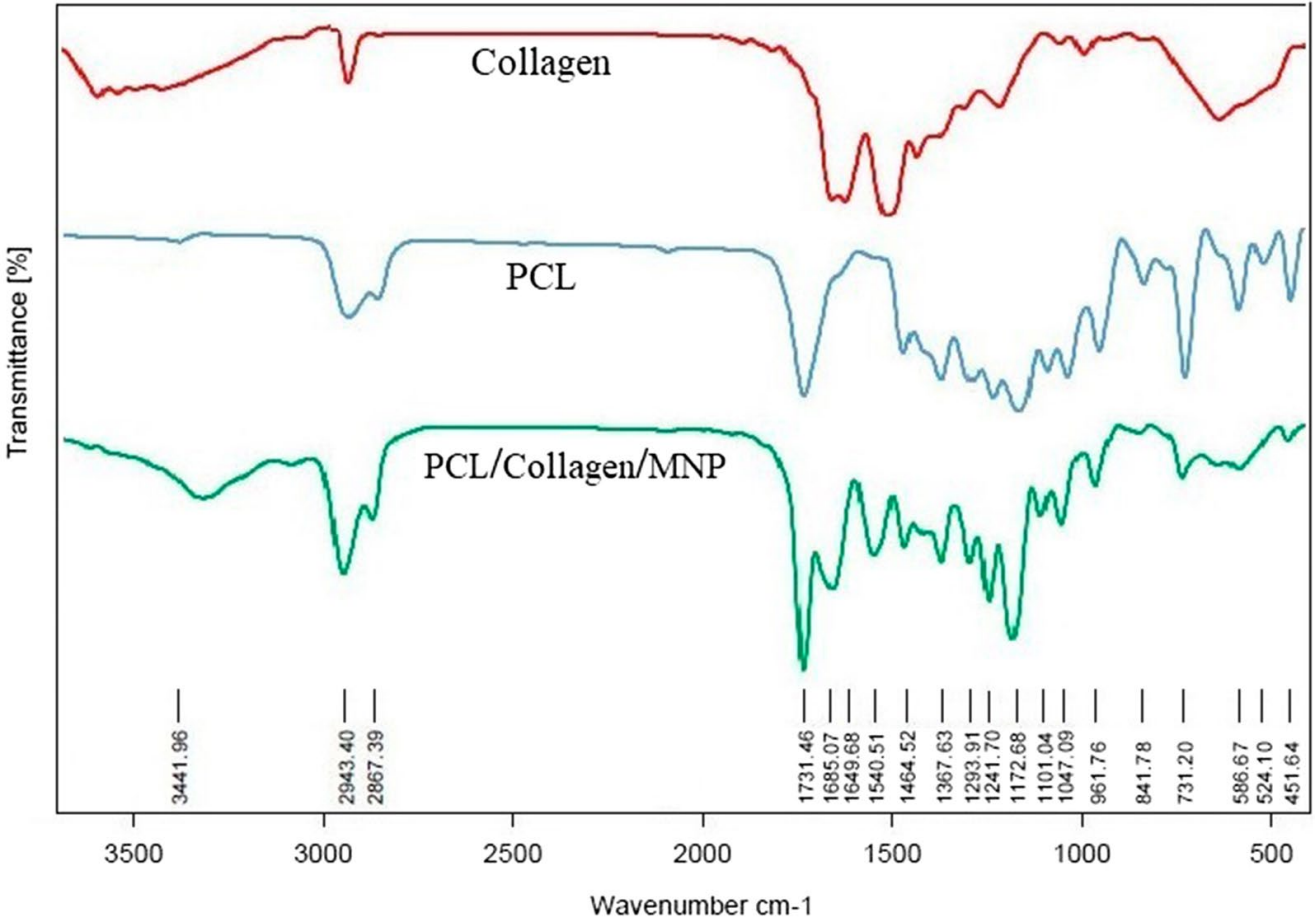
FTIR spectrum of Collagen, PCL, and PCL/Collagen/MNPs scaffolds. Collagen exhibited the presence of four characteristic FTIR peaks including amide groups, which were typical for type I collagen (red graph). PCL polymer indicated the presence of three peaks attributed to CH_2_, C=O, and C-O stretching (blue graph). FTIR spectra of nanocomposite scaffold indicate the main peaks which approved the presence of PCL, collagen and MNPs in the structure of fabricated scaffolds (green graph)

FTIR spectra analysis of natural Col I showed four significant peaks around 3300 cm^−1^, 2900 cm^−1^_,_ 1650 cm^−1^, and 1550 cm^−1^ (Fig. 3, red graph), which is attributed to the stretching vibrations from amide A (Hydrogen-bonded -NH groups), amide B (C–H-bonded –NH groups), polypeptide backbone (C=O stretching), and amide II (N–H bending and C-H stretching), respectively. These results were consistent with FTIR results of calfskin extracted collagen [25]. FTIR spectra analysis of natural PCL showed four significant peaks around 2940 cm^−1^, 2860 cm^−1^, and 1720 cm^−1^ (Fig. 3, blue graph) which is attributed to the stretching vibrations from –CH_2_, C=O, and C–O, respectively [26]. Notably, FTIR spectra analysis of nanocomposite scaffold indicated the main above-mentioned Col I peaks (Fig. 3, green graph), confirming the maintenance of the natural structure of Col I upon electrospinning and scaffold preparation. In addition, this graph showed the PCL and Fe–O peaks which are attributed to the presence of PCL and MNPs in nanocomposite scaffold. Overall, the FTIR analysis supported the structural integrity of Col I, PCL, and MNPs.

**Fig. 4:**
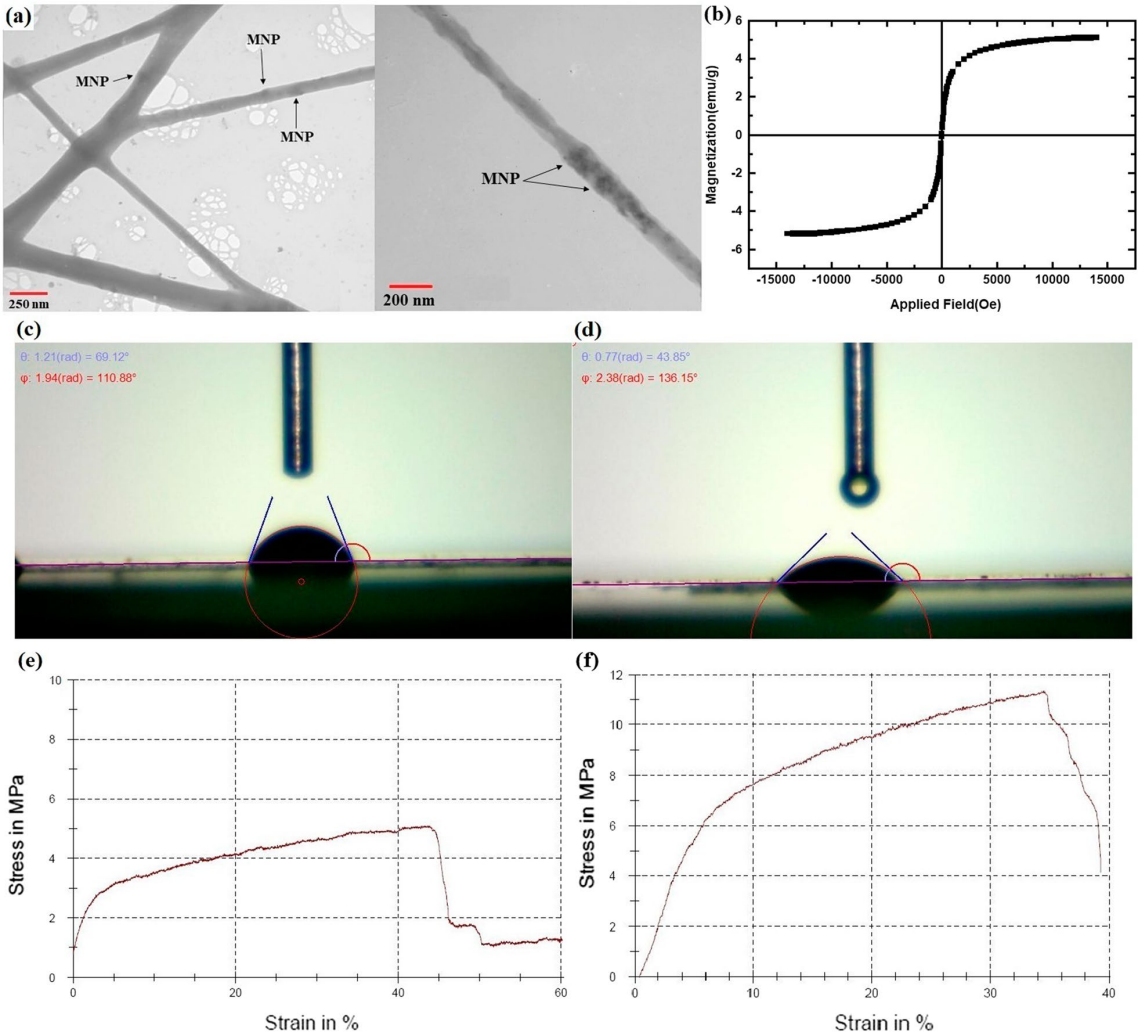
Morphological and biophysical behaviors of nanomagnetic scaffolds. **a** TEM images show the internal structure revealing incorporation of MNPs in the scaffolds nanofiber. **b** Magnetic properties of the nanocomposite scaffolds analyzed by a VSM. **c**, **d** Contact angle analysis was used to evaluate the wettability of the nanocomposite scaffolds, indicating significant improvement the hydrophilicity of the nanomagnetic scaffolds. Stress–strain curves showing the mechanical properties of PCL/Col I scaffold (**e**) and nanomagnetic scaffold (**f**); revealing the enhancement of the tensile strength, upon incorporation of Fe_3_O_4_ into scaffolds structure

More importantly, the incorporation of MNPs in each scaffold fiber presents a nanocomposite scaffold with endogenous magnetic properties as confirmed by TEM cross-sectional images (Fig. 4a). Previous studies have been decorated the surface of scaffolds with MNPs [20, 22]. However, in this study, MNPs are completely located inner the nanofibers with no effects on the porosity of scaffold. The TEM results showed that the MNPs randomly distributed in scaffold's fiber (Fig. 4a) and confirmed MNPs free of agglomeration within the solution are the prerequisite for the electrospinning into nanofibers without bead formation [10].

Moreover, VSM analysis of the nanocomposite confirmed the presence of the MNPs into the magnetoactive scaffolds (Fig. 4b) and revealed a typical ferromagnetic behavior [27]. However, compared to MNPs alone (Fig. 1b), this property is slightly reduced due to the covering of MNPs by fiber-containing polymers upon electrospinning.

The water contact angle was measured to investigate the wettability of the scaffold. Pure PCL scaffold possesses hydrophobic behavior with the contact angle of 114° [28] due to the absence of hydrophilic functional groups in the chemical structure of PCL polymer [29]. However, the presence of Col I in PCL scaffold induced a slight hydrophilic surface, in which contact angle was about 70° (Fig. 4c). After the 7 wt% of hydrophilic Fe_3_O_4_ NPs is added to scaffold, the contact angle was significantly decreased to approximately 43° (Fig. 4d). Besides Col I, MNPs changed the wetting behavior in the both bulk and surface of the scaffold possibly through establishing the hydrogen bond between the oxygen of Fe_3_O_4_ in the scaffolds and water molecules droplets [30]. MNPs enhance the wettability of scaffolds which aimed to provide cell attachment, nutrition, and growth [21, 30].

The tensile strengths obtained for the PCL/Col I scaffold and nanomagnetic scaffold were 5.09 MPa and 11.33 MPa, respectively (Fig. 4e, f). In addition, the total elongation for PCL/Col I scaffold and nanomagnetic scaffold was 44.29% and 33.97%, respectively (Fig. 4e, f). It was observed that an entrapped of MNPs into nano-scaffold led to an increase the tensile strength, while its elongation was slightly reduced. Indeed, homogenous dispersion of metal-based MNPs acted as the backbone and improved the mechanical strength of nanocomposite scaffold along with high porosity [31]. In addition, the Col I network produces the thinner bridges between PCL polymers (Fig. 2), and hydrogen bonds between the helix in the triple helix of Col I molecule could increase internal strength as well as scaffold stability [32]. Another notable influence of the MNPs was observed in the tensile mechanical properties. However, the concentration of MNPs in nanofibers influenced the scaffold resistance to tensile strength or deformation as well as its stiffness, which should be considered upon preparation of scaffolding materials [10]. Overall, these results highlight the key role of MNPs for demonstrating in situ magnetism in polymeric scaffolds and also introducing strong interfacial interaction between the components of nanocomposite constructs for their applicability in BTE.

### ADSCs identification, scaffold biocompatibility, and cell adhesion

**Fig. 5:**
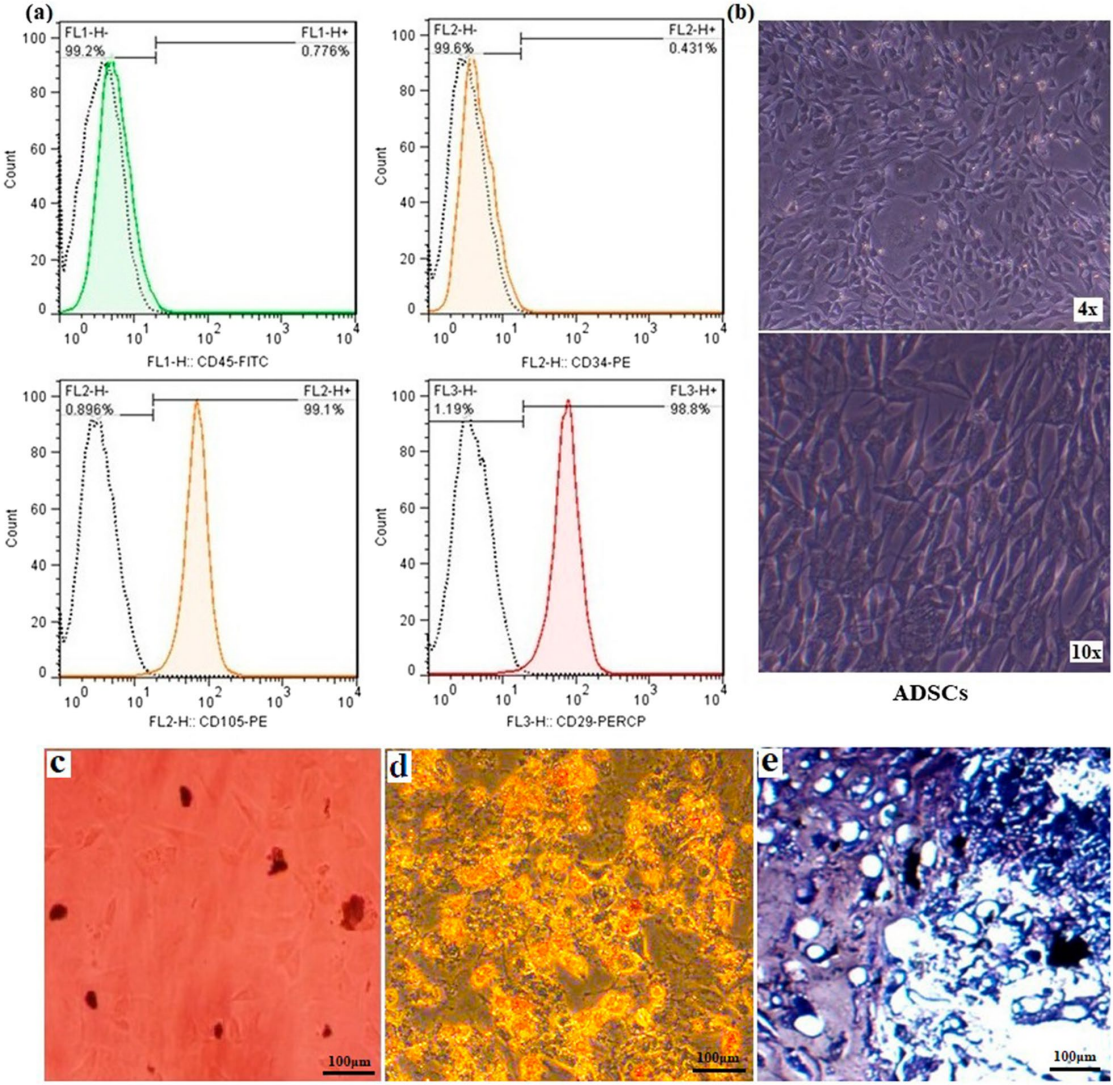
ADSCs characterization **a** Flow cytometry analysis of rat ADSCs was positive for CD105 and CD29 and negative for CD34 and CD45. **b** Inverted microscopic imaging at different resolutions shows an elongated fibroblast-like morphology of extracted rat ADSCs. **c–e** In vitro multilineage differentiation of ADSCs, including **c** Osteogenic differentiation, **d** Adipogenic differentiation, and **e** chondrogenic differentiation

ADSCs cells present few ethical concerns, and subcutaneous fat has become an ideal tissue source for these cells for regenerative medicine [33]. In this study, rat-sourced ADSCs were used to assess cell-scaffold compatibility/attachment and the osteogenesis potential of the scaffolds in vitro. At first, the flow cytometry assay was used to identify the undifferentiated ADSCs phenotype according to their surface markers. As presented in Fig. 5a, about 98.8% and 99.1% of extracted cells were strong positivity for CD29 and CD105, respectively, while 99.6% and 99.2% of those cells were negative for hematopoietic CD34 and CD45 markers. These results confirmed the purity of isolated ADSCs [34]. In addition, the morphology of isolated cells (spindle shape) was observed by inverted microscopic imaging (Fig. 5b). Three lineage differentiation potential of ADSCs was assessed using three different staining methods. Red-stained mineral calcium deposits were evidenced by Alizarin Red S under osteogenic culture conditions (Fig. 5c), whereas adipo-inductive medium induced the production of intracellular red-colored lipid droplets after staining with Oil Red (Fig. 5d). Moreover, we showed that chondro-inductive medium resulted in chondrogenic differentiation of the cells as evidenced by the synthesis of blue-stained proteoglycan aggrecan via Alcian blue staining (Fig. 5e). Our experiments revealed that isolated ADSCs can give rise to several mesenchymal lineages.

**Fig. 6:**
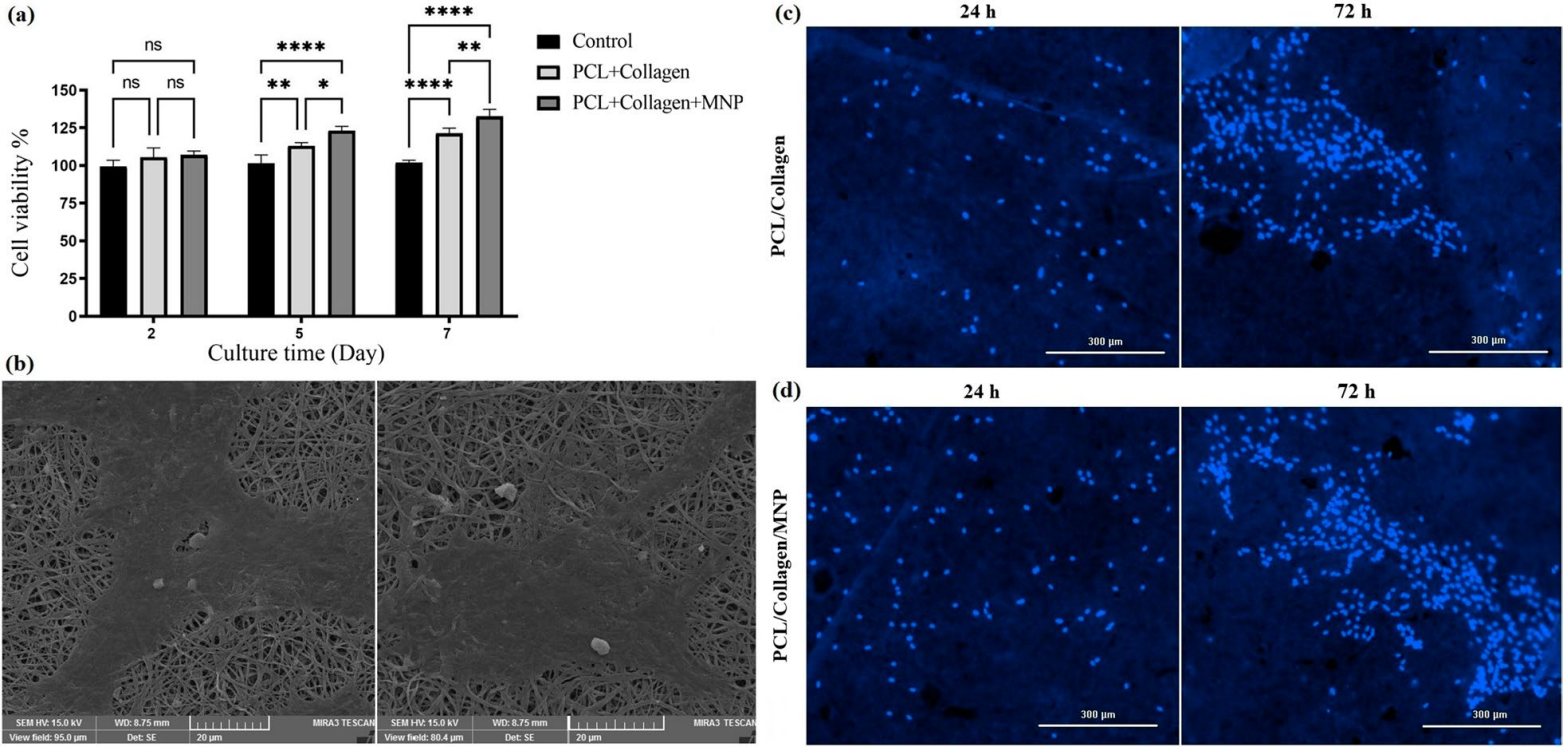
**a** Cell viability of ADSCs seeding on the nanofibrous scaffolds with and without the MNPs by MTT assay for 2, 5, and 7 days. **b** SEM imaging of ADSCs attachment and spreading on the nanomagnetic scaffolds. **c**, **d** Fluorescence micrographs of DAPI staining of living ADSCs cells on PCL/Col I and MNPs + PCL/Col I scaffolds for 24 and 72 h

The effects of PCL/Col I and MNPs + PCL/Col I scaffolds on cells viability of each experimental group were examined by MTT assay (Fig. 6a). The non-cytotoxicity and cell compatibility related to MNPs-loaded scaffold are observed compared to the control (i.e., ASCDs culture without scaffold). Both of the basic and nanocomposite scaffolds induced the proliferation of seeded cells over time (5–7 days) (Fig. 6a), which confirm the biocompatible nature of these scaffolds and safety of FDA-approved PCL [29] and Fe_3_O_4_ [19] for clinical applications.

The cell adhesion onto the nanocomposite scaffolds is the first fundamental step to determine whether the used materials could be applied as scaffolds for BTE [7, 8]. SEM imaging and DAPI staining showed the spreading and morphology of seeded ADSCs on the surface of nanofibrous scaffolds (Fig. 6b–d). DAPI staining demonstrated that the nuclei of living cells have a normal shape at 24 h and 72 h (Fig. 6c, d). Furthermore, SEM imaging confirmed that the seeded cells were firmly attached and even spread into macropores of nanocomposite fiber structure (Fig. 6b). DAPI staining also showed the proliferation of seeded ADSCs on PCL/Col I after 72 h (Fig. 6c), and this finding was more significant in MNPs + PCL/Col I scaffolds (Fig. 6d). Thus, along with suitable topography (Fig. 2g, h) and wettability of the nanocomposite scaffolds (Fig. 4c, d), MNPs also are able to provide a suitable biophysical microenvironment to improve scaffold cytocompatibility as well as increase the cells adherence and proliferation [35]. In the present study, the proliferation of ADSCs on different scaffolds (Fig. 6a, c, d) indicated that the incorporation of nanoparticles influenced the cellular adhesion and induced the whole growth process in seeded cells. SEM results indicated that the macropores of fabricated biocompatible nanocomposite scaffolds are in the range of 70–150 μm, in which scaffolds with 120 µm pore size revealed the best cell attachment [21].

### Osteo-differentiation of ADSCs

**Fig. 7:**
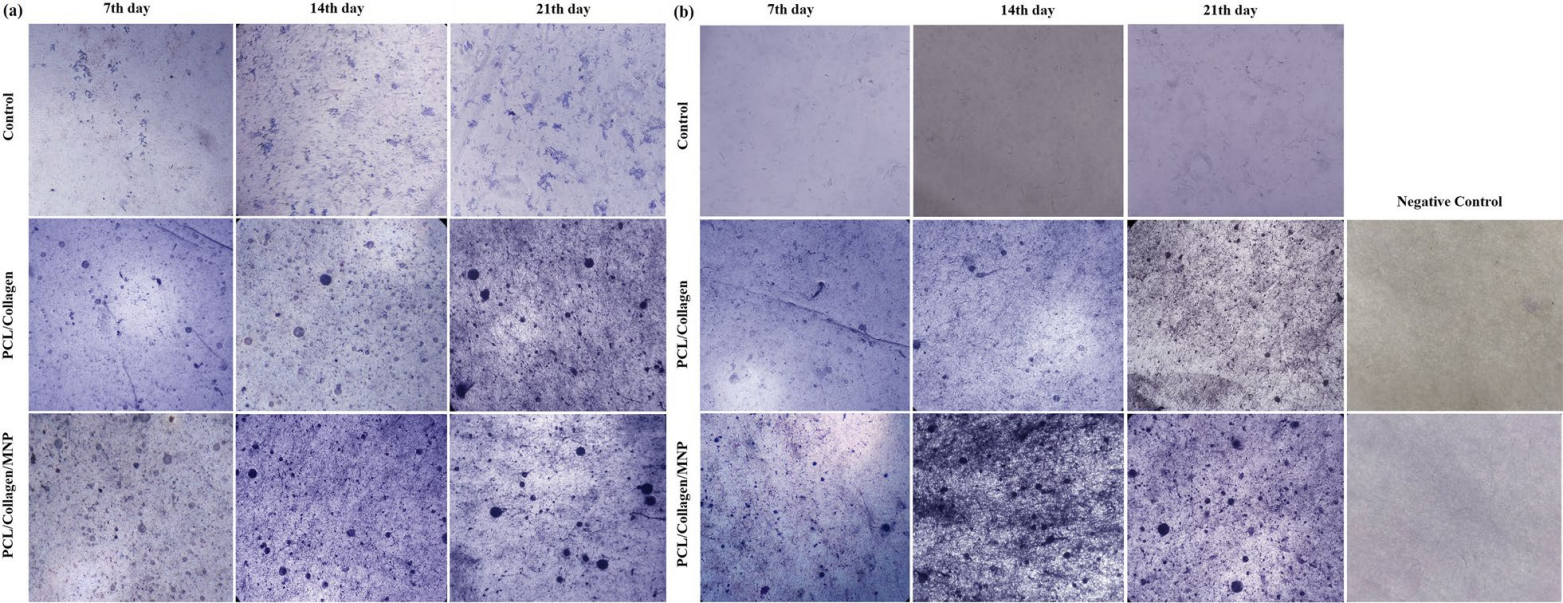
ALP activity of cultured ADSCs (control), ADSCs-seeded PCL/Col I scaffolds, and ADSCs-seeded MNPs + PCL/Col I scaffolds after 7, 14, and 21 days in with (**a**), and without (**b**) osteogenic media condition as indicated by a purple appearance

The osteo-induction capacity of the nanocomposite scaffold was investigated by ALP activity as an indicator of early‐stage bone cell differentiation [36]. ALP activity was indicated by microscopic images of purple spots inside cells at days 7, 14, and 21 (Fig. 7a). Based on data, ALP activity was raised on the 7th day among the three groups and compared to the control group or PCL/Col scaffold, the ALP spots have increased in MNPs-loaded scaffolds. The local physical stimulus introduced by whole magnetic nanoparticles into the scaffold improved the stem cell's differentiation [37]. Over time (7–21 days), the enzyme activity was increased in all groups, and this indicator reached maximum levels, especially in MNP-modified scaffold-seeded cells. Static magnetic field (SMF) is well documented as an inducer of stem cells differentiation [38]; however, in this study, the endogenous magnetic field produced by the existence of a geomagnetic field promoted the stem cells- osteogenic differentiation and osteogenesis [17].

As expected, when the osteoinductive medium was used, the ADSCs differentiation and enhancement of enzyme activity were observed (Fig. 7a). However, the number of purple spots related to ALP activity was rarely observed in cultured ADSCs in osteogenic cues-free media conditions over 7–21 days (Fig. 7b). The ALP activity was observed in osteogenic cues-free media conditions in both scaffolds. In addition, the ALP activity was more evident in the magnetic scaffolds compared to the PCL/Col scaffolds as shown in the increase in the number and area of purple spots (Fig. 7b). This method reduces the price attributed to osteogenic media and also declined the possible contamination with non-human pathogens, host immune responses, and uncountable effects of this media on stem cell differentiation [39]. Moreover, the short half-life of media growth factors resulted in the rapid loss of their functions in vivo [5]. In our experiments, the MNPs-loaded scaffold introduces a bioactive construct in osteogenic cue-free media conditions.

**Fig. 8:**
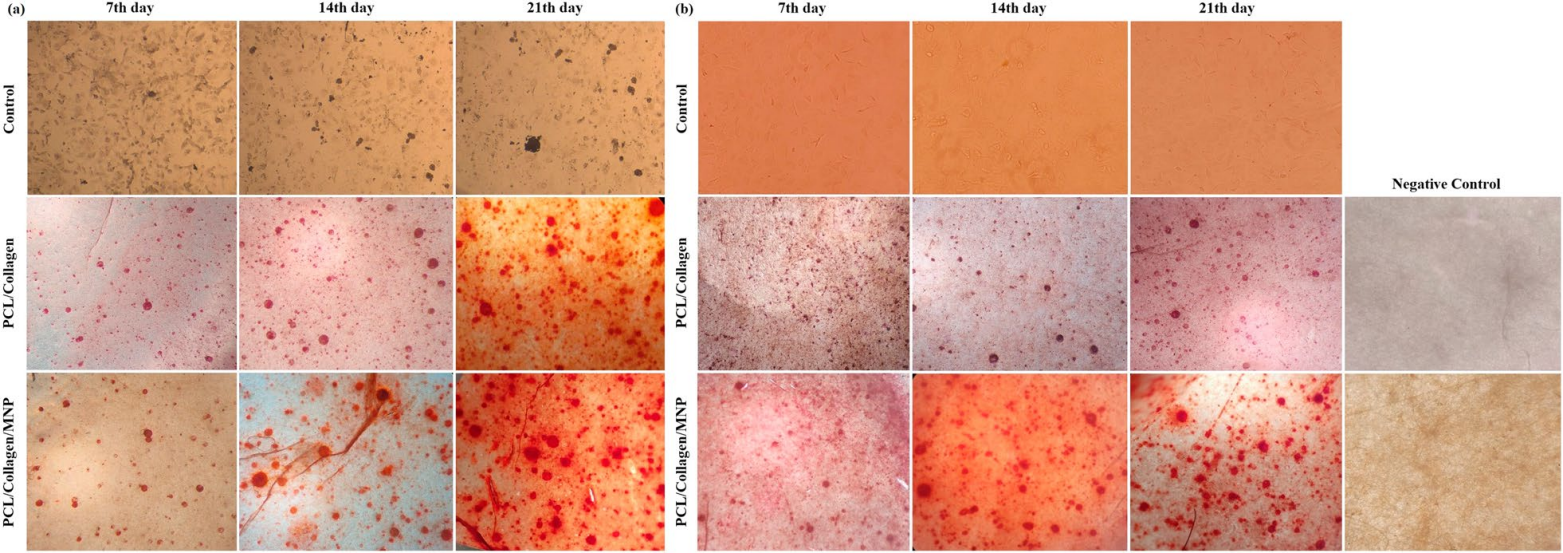
Microscopic images of Alizarin red S staining of cultured ADSCs (control), ADSCs-seeded PCL/Col I scaffolds, and ADSCs-seeded MNPs + PCL/Col I scaffolds after 7, 14, and 21 days in with (**a**), and without (**b**) osteogenic media condition as indicated the calcium deposition as dark red spots appearance

Typically, the matrix mineralization is evaluated by Alizarin red S staining to confirm the deposition of calcium in vitro as identified by dark red spots. Figure 8a has shown that compared to PCL/Col I scaffold, MNPs significantly enhanced the calcium deposition ability of ADSCs seeded in the PCL/Col I scaffold over 7–21 days. The Alizarin red S staining data reflect calcium-mineralization upon cell proliferation and differentiation since this indicator was non-significant in cultured ADSCs alone for 7–21 days. Compared to ADSCs alone, the number of dark red spots related to calcium content was more evident after 7–21 days of incubation in fabricated scaffolds (Fig. 8a). These results have shown the mineralization effect of these scaffolds, as a recommended feature for BTE scaffolds [40]. The calcium deposition was observed in both scaffolds, and this indicator in the magnetic scaffolds was higher than the MNPs-free scaffolds. Similar to the results obtained with ALP activity, calcium depositions were observed in PCL/Col I and MNPs + PCL/Col I scaffolds in osteogenic cues-free media (Fig. 8b). In the absence of osteogenic media, both PCL/Col I and MNPs containing scaffolds promoted the osteogenesis and deposition of calcium in a significant ratio, while this indicator was not detected with ADSCs alone over the same period (Fig. 8b). Notably, a high concentration of serum protein will inhibit calcium phosphate crystallization and bone formation [41], thus, osteogenic cues-free media conditions declined this drawback. These results indicated that seeded ADSCs subjected to MNPs are able to migrate into the inner region of the conductive scaffolds as well as increase calcium content in the scaffolds toward bone formation.

**Fig. 9:**
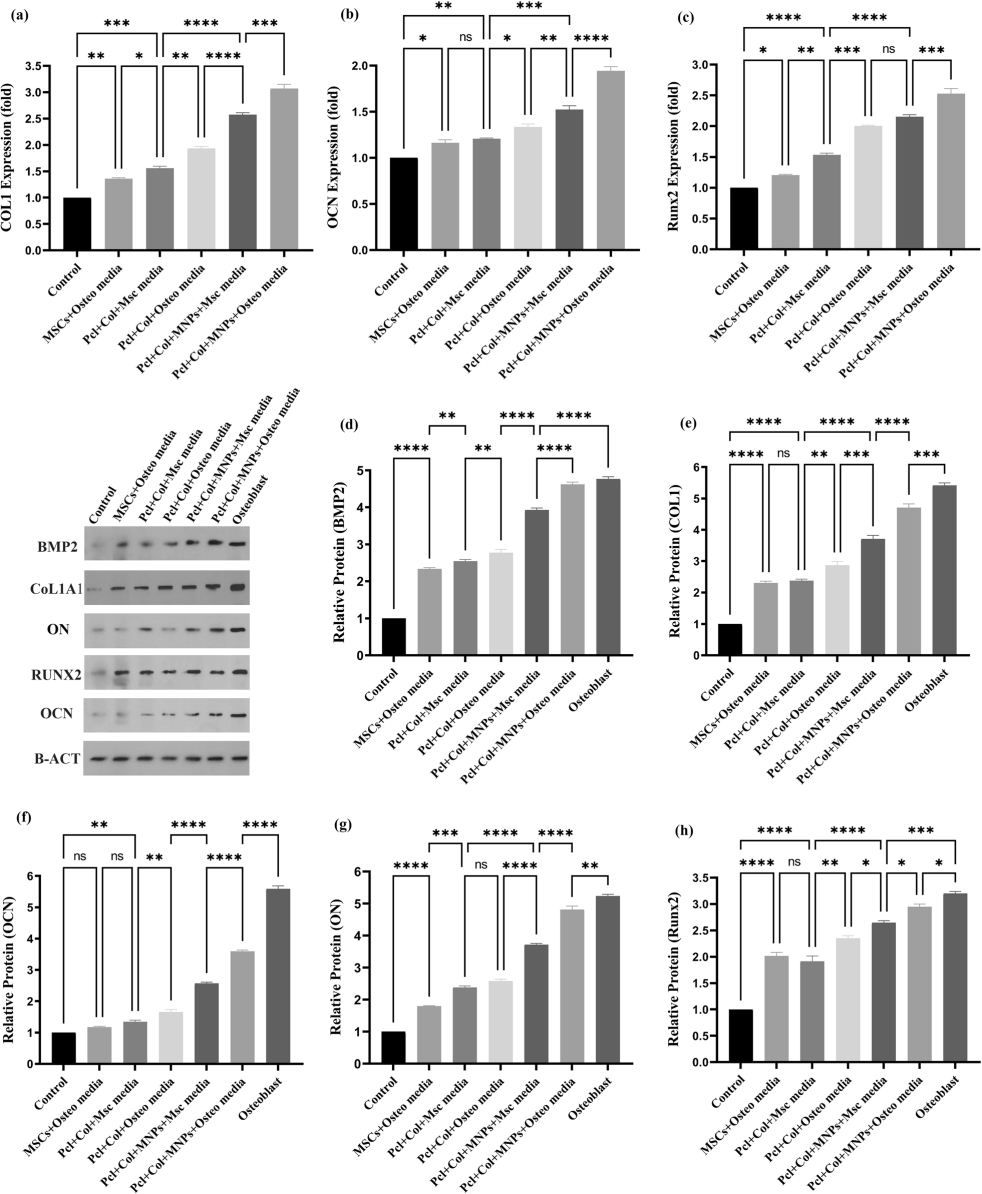
Effects of the MNPs + PCL/Col I and PCL/Col I scaffolds on the osteogenic differentiation of ADSCs seeding on scaffolds. **a–c** Gene expressions of COL1, OCN and Runx2 by qRT-PCR. **d–h** Protein level of Col I, OCN, Runx2, BMP2, and ON proteins by Western blot analysis. Both of assays performed on seeded ADSCs at 21 days of incubation in osteo and osteo cues-free media conditions. Data are the representative of three independent experiments. One-Way ANOVA with Tukey post hoc analysis. ∗*p* < 0.05, ∗∗*p* < 0.01, ∗∗∗*p* < 0.001, and ∗∗∗∗*p* < 0.0001. Negative control (ADSCs alone whiteout osteo media); Positive control (Osteoblast cells)

Finally, the effect of MNP-modified scaffolds on cellular behavior at molecular level was examined using qRT-PCR to measure some important osteogenesis-related genes. Compared to control (i.e., ADSCs or ADMSCs cells) and PCL/Col I groups, the mRNA expressions of Col I, OCN, and Runx2 significantly enhanced in MNPs + PCL/Col I group after 21 days of incubation in osteo and osteo cues-free media conditions (Fig. 9a–c). The expression levels of these genes in cells on the PCL/Col I and MNP-modified scaffolds were markedly upregulated, and this effect was augmented with the addition of osteogenic media. According to the qRT-PCR results, the nanocomposite scaffolds could greatly enhance the expression of Col I in presence and absence of osteogenic media condition (Fig. 9a). Higher expression of COL I could be attributed to the rigidity of nanocomposite surface as was more than that of non-magnetic ones [17]. The geomagnetic field provides magnetic stimulation for MNPs [17], resulting in mechanical vibration of MNPs promoting the piezoelectricity of Col I [42, 43]. The nature of piezoelectric properties of Col I has been found to provide electromechanical cues to be effective in enhancing rat MSC/ADSCs migration, proliferation, and differentiation in vivo [44, 45].

Osteocalcin (OCN) is solely an osteoblasts-secreted protein and is considered a marker of osteogenic differentiation in late stage [46]. Significant upregulation of OCN expression was found in the MNPs-loaded scaffold, compared to the nan-magnetic scaffolds or control cells groups (Fig. 9b). Runx2 is the osteogenic master gene that acts as a transcription factor for osteoblast differentiation and bone formation [20]. The aims of gene therapy, the immobilization of Runx2 plasmid-loaded liposomes at the surface of electrospun PCL induced osteogenic differentiation in BMSCs in vitro [47], in which overexpressed Runx2 subsequently upregulates OCN driving of BMSCs toward osteogenic differentiation and bone healing [15]. Thus, for further validation of osteogenesis, the mRNA expression of Runx2 was quantified. The results showed that the expression of this gene is significantly increased while cells seeded on the magnetic scaffolds (Fig. 9c). As shown in Fig. 9a–c, the expression of COL I, OCN, and Runx2 was upregulated in both PCL/Col I and MNP-modified scaffolds incubated in the presence and absence of osteo media. qRT-PCR results indicated that MNPs increased expression levels of multiple genes involved in bone differentiation.

As the increased expression of those genes might regulate both mineralization and transcription in ADSCs into the MNPs-modified bioactive scaffolds, we postulate that the spontaneous osteo-differentiation can be attributed to the proteins expressed by these genes. The western blotting assay demonstrated that these scaffolds, especially magnetic ones, upregulated the expression of Col I (Type I collagen), OCN (Osteocalcin), Runx2 (Runt-related transcription factor 2), BMP2 (Bone morphogenetic protein 2), and ON (Osteonectin) proteins in seeded ADSCs at 21 days of incubation in osteo and osteo cues-free media conditions (Fig. 9d–h). Nonetheless, the expressions of these proteins in ADSCs seeded on nanomagnetic scaffolds incubated with osteogenic and osteogenic cues-free media conditions were near to osteoblast cells as a positive control. Moreover, the levels of BMP-2 protein were increased greatly by the combined magnetic actuation (MNPs + PCL/Col I *vs.* PCL/Col I), and the potential role of these scaffolds was revealed even in a medium free of osteogenic supplementation. Osteonectin as an extracellular matrix glycoprotein is secreted by osteoblasts in the mid-stage of bone formation and mediated cell–matrix interaction, initiation of mineralization, formation of mineral crystal, and Col I binding [48, 49]. The mineralization function of osteonectin is indicated by induction of the deposition of nanohydroxyapatite on Col I along with bone development [50]. This set of western blotting findings confirmed the qRT-PCR observations and implied that polymeric scaffolds-loaded MNPs accelerated ADSCs differentiation due to the ability of bone tissue to recognize mechano-electrical conversion [20, 22]. Altogether, the present approach, endogenous magnetic scaffolding indicated a potential tool to stimulate ADSCs differentiation in vitro even in osteogenic cues-free media conditions. However, orthotopic transplantation of this magnetic nanocomposite into a bone defect rat model will be a necessary further step to test the implementation of the present construct toward clinical applications.

From a molecular pathway viewpoint, the MNPs incorporated with PCL/Col I nanofibrous scaffold can provoke several signaling biomolecules associated with different osteogenic-related signaling pathways. In this regard, it has been shown that an increased BMP-2 expression, belonging to the TGF-β family, can result in the activation of Smad proteins such as Smad 2, 3, and 4 and osteogenesis-specific factors Runx2, leading to promoted osteoblast differentiation [15, 20, 36]. In addition, BMP2 interacts with the Wnt/β-catenin pathway results in promoting the osteogenic differentiation and maintenance of osteoblast progenitors and bone homeostasis [51]. Several factors related to Wnt signaling such as Wnt3a, 4, 5a, 7b, 10b, and 16 in collaboration with the mTOR/JNK/c-Jun axis can promote the expression of osteogenesis-related genes [52]. Considering the existence of crosstalk between TGF-β family with other signaling cascades, it was also mentioned that the incorporation of MNPs into scaffolds can induce the mitogen-activated protein kinase (MAPK) pathway-related effectors such as MEK1, 4, c-JUN and, JunD factors. Along with these changes, downstream genes, i.e., BMP2 and Runx2 are upregulated. Moreover, the interaction of cell membrane and MNPs might be produced mechanical stress signals to stimulate the osteogenic differentiation of stem cells [15]. In contrast to these findings, inactivation of the extracellular signal-regulated kinase 1 (ERK1) and ERK2 resulted in downregulation of β-catenin and activation of the alternative chondrogenic pathway, suggesting a role for canonical Wnt signaling in supporting osteoblast formation [53]. These data demonstrated that the exposure of ADSCs to MNPs inspires osteoblast-like activity via provoking an array of intracellular effectors in different steps of differentiation procedure.

There are some limitations in this study that need more consideration. It is suggested that future studies should investigate an in vivo regenerative outcome of the used scaffold in the context of bone regeneration. We also did not measure the transcription levels of some genes related to specific signaling pathways such as Ras, kinase activity along with the expression of associated factors like MAPK, Wnt.

## Conclusion

Herein, we developed a robust strategy to fabricate a bioactive nanocomposite construct utilizing Fe_3_O_4_ entrapped in PCL/Col I scaffold and determined whether it is beneficial to osteogenic differentiation for potential applications in BTE. The MNPs-loaded scaffolds were fabricated using the electrospinning process, yielding a spatially uniform distribution of MNPs inner the roughed structure as SEM, TEM, and AFM indicated. Similar to Col I, MNPs improved the scaffold’s hydrophilicity and stability with no effects on the porosity of the scaffold. ADSCs seeded on the nanocomposite showed suitable affinity and spreading, and even the fabricated scaffolds slightly increased ADSCs proliferation. Furthermore, these biocompatible MNPs-loaded scaffolds exhibited endogenous magnetic properties that can induce the osteogenic differentiation of ADSCs by stimulating osteogenic-related signaling pathways and providing a suitable microenvironment for osteogenesis. This phenomenon was initially demonstrated by significantly elevated osteogenic markers such as ALP activity and calcium deposition, even in osteogenic cues-free media conditions. The nanocomposite scaffolds upregulated the expression of representative genes and proteins (i.e., BMP2, Col I, Runx2, OCN, and ON) regarding osteogenesis. The MNP-modified scaffolds possess biocompatible and bioactive properties to promote the spontaneous osteogenic differentiation of ADSCs in vitro. However, the functionality of this construct requires further investigation in the rat model as aimed to study by our teamwork in the future. We envision that applying a cost-effective magnetic scaffold allows us to craft a broad range of strategies for regenerative medicine.

## Data Availability

The data that support the findings of this study are available from the corresponding author upon reasonable request.

## Abbreviations

ADSCs: Adipose-derived stem cells
ARS: Alizarin red staining
ALP: Alkaline phosphatase
BTE: Bone tissue engineering
qRT-PCR: Quantitative real-time polymerase chain reaction
GAPDH: Glyceraldehyde-3-phosphate dehydrogenase
RUNX2: Runt-related transcription factor 2
Col1: Collagen type I
MNP: Magnetic nanoparticle
PCL: Polycaprolactone
OCN: Osteocalcin
ON: Osteonectin
